# (2*E*)-1-(2,6-Dichloro-3-fluoro­phen­yl)-3-(4-meth­oxy­phen­yl)prop-2-en-1-one

**DOI:** 10.1107/S1600536812011841

**Published:** 2012-03-24

**Authors:** A. S. Praveen, Jerry P. Jasinski, James A. Golen, H. S. Yathirajan, B. Narayana

**Affiliations:** a Department of Studies in Chemistry, University of Mysore, Manasagangotri, Mysore 570 006, India; b Department of Chemistry, Keene State College, 229 Main Street, Keene, NH 03435-2001, USA; c Department of Studies in Chemistry, Mangalore University, Mangalagangotri, 574 199, India

## Abstract

There are two independent mol­ecules in the asymmetric unit of the title compound, C_16_H_11_Cl_2_FO_2_. The F atom equally populates both *meta* positions of the 6-dichloro-3-fluoro­phenyl ring in each mol­ecule, resulting in 0.5 occupancy for both the F and H atoms in these positions. The dihedral angle between the mean planes of the benzene rings are 77.5 (2) and 89.8 (8)°in the two mol­ecules. In the crystal, weak C—H⋯F and C—H⋯O inter­actions involving the half-occupied H and F atoms are observed. Weak π–π stacking inter­actions [centroid—centroid distance = 3.150 (2) Å] also contribute to the crystal stability.

## Related literature

For the pharmacological importance of chalcones, see: Dominguez *et al.* (2001 ▶); Li *et al.* (1995 ▶); Mei *et al.* (2001 ▶); Sarojini *et al.* (2006 ▶). For related structures, see: Betz *et al.* (2012 ▶); Yathirajan *et al.* (2007 ▶). For standard bond lengths, see: Allen *et al.* (1987 ▶).
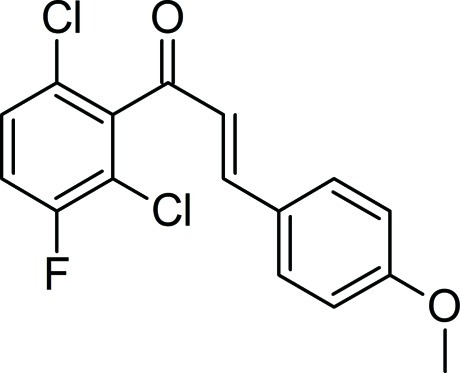


## Experimental

### 

#### Crystal data

C_16_H_11_Cl_2_FO_2_
*M* *_r_* = 325.15
Monoclinic, 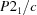

*a* = 11.9035 (6) Å
*b* = 10.4472 (5) Å
*c* = 23.7435 (12) Å
β = 92.296 (4)°
*V* = 2950.3 (3) Å^3^
*Z* = 8
Mo *K*α radiation
μ = 0.45 mm^−1^
*T* = 173 K
0.24 × 0.20 × 0.17 mm

#### Data collection

Oxford Diffraction Xcalibur Eos Gemini diffractometer
Absorption correction: multi-scan (*CrysAlis RED*; Oxford Diffraction, 2010 ▶) *T*_min_ = 0.900, *T*_max_ = 0.927
15257 measured reflections
7015 independent reflections
5165 reflections with *I* > 2σ(*I*)
*R*_int_ = 0.023

#### Refinement

*R*[*F* ^2^ > 2σ(*F* ^2^)] = 0.061
*wR*(*F* ^2^) = 0.145
*S* = 1.06
7015 reflections
401 parameters
4 restraints
H-atom parameters constrained
Δρ_max_ = 0.67 e Å^−3^
Δρ_min_ = −0.57 e Å^−3^

### 

Data collection: *CrysAlis PRO* (Oxford Diffraction, 2010 ▶); cell refinement: *CrysAlis PRO*; data reduction: *CrysAlis RED* (Oxford Diffraction, 2010 ▶); program(s) used to solve structure: *SHELXS97* (Sheldrick, 2008 ▶); program(s) used to refine structure: *SHELXL97* (Sheldrick, 2008 ▶); molecular graphics: *SHELXTL* (Sheldrick, 2008 ▶); software used to prepare material for publication: *SHELXTL*.

## Supplementary Material

Crystal structure: contains datablock(s) global, I. DOI: 10.1107/S1600536812011841/bt5846sup1.cif

Structure factors: contains datablock(s) I. DOI: 10.1107/S1600536812011841/bt5846Isup2.hkl

Supplementary material file. DOI: 10.1107/S1600536812011841/bt5846Isup3.cml

Additional supplementary materials:  crystallographic information; 3D view; checkCIF report

## Figures and Tables

**Table 1:** Hydrogen-bond geometry (Å, °)

*D*—H⋯*A*	*D*—H	H⋯*A*	*D*⋯*A*	*D*—H⋯*A*
C2—H2*A*⋯F1*A*^i^	0.95	2.79	3.657 (7)	153
C4—H4*A*⋯F1^ii^	0.95	2.75	3.410 (5)	127
C11—H11*A*⋯O3^ii^	0.95	2.56	3.451 (3)	156

Symmetry codes: (i) 
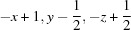
; (ii) 
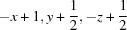
.

**Fig. 1.**
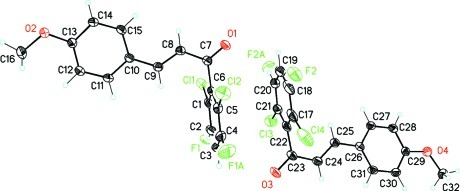
Molecular structure of the title compound showing the atom labeling scheme and 50% probability displacement ellipsoids for two molecules in the asymmetric unit.

**Fig. 2.**
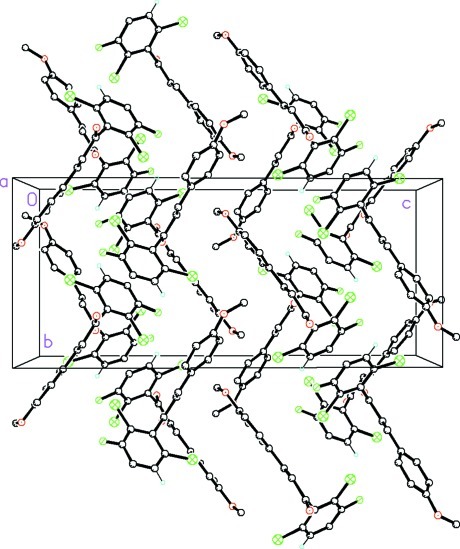
Packing diagram of the title compound viewed along the a axis.

## supplementary crystallographic information

### Comment

Many Chalcones are known to exhibit various biological properties such as antimalarial (Li *et al.*, 1995), antifungal (Dominguez *et al.*, 2001) and antibacterial activity (Mei *et al.*, 2001). They are also finding application as organic nonlinear optical materials (NLO) for their SHG conversion efficiency (Sarojini *et al.*, 2006). Crystal structures of some related chalcones, *viz*., (2E)-1-(2,4-dichlorophenyl)-3-(2-hydroxy-3-methoxyphenyl)prop-2-en-1-one (Yathirajan *et al.*, 2007) and (2E)-1-(2,6-dichloro-3-fluorophenyl)-3-(4-fluorophenyl)prop-2-en-1-one (Betz *et al.*, 2012) have been reported. As part of our ongoing studies on chalcones, the title compound (I), C_16_H_11_C_l2_FO_2_, was synthesized and its crystal structure is reported.

In (I) two molecules crystallize in the asymmetric unit (Fig. 1). In the 2,6-dichloro-3-fluorophenyl ring, the fluorine atom equally populates both *meta* positions of the phenyl ring in each molecule (C2 & C4; C18 & C20) resulting in 0.5 occupancy for both the fluorine and hydrogen atoms (H2A & H4A; H18A & H20A) in these positions. The dihedral angle between the mean planes of the benzene rings in each molecule is 77.5 (2)° and 89.8 (8)°, respectively. Bond lengths are in normal ranges (Allen *et al.*, 1987). Crystal packing is enhanced by weak C—H···F and C—H···O intermolecular interactions (Table 1) from both half-occupied H and F atoms supporting parallel chains along the *b* axis (Fig. 2) aa well as weak π–π stacking interactions (Table 2).

### Experimental

To a stirred solution of 1-(2,6-dichloro-3-fluorophenyl)ethanone (1 g, 4.8 mmol) and 4-methoxybenzaldehyde (0.65 g, 4.8 mmol) in ethanol (10 ml), powdered KOH (0.40 g, 7.2 mmol) was added at 273 K. The reaction mixture was stirred at room temperature for 3 h. After completion of the reaction, the reaction mixture was poured to ice cold water and acidified with 1.5 N HCl (pH _3_). The solid precipitated was filtered and dried to afford 1.4 g of the title compound, (I,) in 89% yield. X-ray quality crystals were obtained by slow evaporation of a tetrahydrofuran solution (m.p.: 361–362 K).

### Refinement

All of the H atoms were placed in their calculated positions and refined using the riding model with C—H lengths of 0.95 Å (CH) or 0.98 Å (CH_3_). The isotropic displacement parameters for these atoms were set from 1.19 to 1.20 (CH), or 1.49 (CH_3_) times *U*_eq_ of the parent atom. Overlapping of the F atoms in the *meta* position of the phenyl ring in each molecule resulted in H2A, H4A, H18A, H20A and F1, F1A, F2, F2A being refined at 0.50 occupancy. C2—F1 and C4—F1A bond distances were fixed at 1.33 (2) Å, C18—F2 and C20—F2A bond distances were fixed at 1.33 (06) Å.

### Crystal data

**Table:**

C_16_H_11_Cl_2_FO_2_	*F*(000) = 1328
*M**_r_* = 325.15	*D*_x_ = 1.464 Mg m^−^^3^
Monoclinic, *P*2_1_/*c*	Mo *K*α radiation, λ = 0.71073 Å
Hall symbol: -P 2ybc	Cell parameters from 3879 reflections
*a* = 11.9035 (6) Å	θ = 3.1–30.0°
*b* = 10.4472 (5) Å	µ = 0.45 mm^−^^1^
*c* = 23.7435 (12) Å	*T* = 173 K
β = 92.296 (4)°	Block, colorless
*V* = 2950.3 (3) Å^3^	0.24 × 0.20 × 0.17 mm
*Z* = 8	

### Data collection

**Table:**

Oxford Diffraction Xcalibur Eos Gemini diffractometer	7015 independent reflections
Radiation source: Enhance (Mo) X-ray Source	5165 reflections with *I* > 2σ(*I*)
Graphite monochromator	*R*_int_ = 0.023
Detector resolution: 16.1500 pixels mm^-1^	θ_max_ = 27.9°, θ_min_ = 3.1°
ω scans	*h* = −10→15
Absorption correction: multi-scan (*CrysAlis RED*; Oxford Diffraction, 2010)	*k* = −13→10
*T*_min_ = 0.900, *T*_max_ = 0.927	*l* = −30→31
15257 measured reflections	

### Refinement

**Table:**

Refinement on *F*^2^	Primary atom site location: structure-invariant direct methods
Least-squares matrix: full	Secondary atom site location: difference Fourier map
*R*[*F*^2^ > 2σ(*F*^2^)] = 0.061	Hydrogen site location: inferred from neighbouring sites
*wR*(*F*^2^) = 0.145	H-atom parameters constrained
*S* = 1.06	*w* = 1/[σ^2^(*F*_o_^2^) + (0.042*P*)^2^ + 3.2887*P*] where *P* = (*F*_o_^2^ + 2*F*_c_^2^)/3
7015 reflections	(Δ/σ)_max_ < 0.001
401 parameters	Δρ_max_ = 0.67 e Å^−^^3^
4 restraints	Δρ_min_ = −0.57 e Å^−^^3^

### Special details

**Table:**

Geometry. All e.s.d.'s (except the e.s.d. in the dihedral angle between two l.s. planes) are estimated using the full covariance matrix. The cell e.s.d.'s are taken into account individually in the estimation of e.s.d.'s in distances, angles and torsion angles; correlations between e.s.d.'s in cell parameters are only used when they are defined by crystal symmetry. An approximate (isotropic) treatment of cell e.s.d.'s is used for estimating e.s.d.'s involving l.s. planes.
Refinement. Refinement of *F*^2^ against ALL reflections. The weighted *R*-factor *wR* and goodness of fit *S* are based on *F*^2^, conventional *R*-factors *R* are based on *F*, with *F* set to zero for negative *F*^2^. The threshold expression of *F*^2^ > σ(*F*^2^) is used only for calculating *R*-factors(gt) *etc*. and is not relevant to the choice of reflections for refinement. *R*-factors based on *F*^2^ are statistically about twice as large as those based on *F*, and *R*- factors based on ALL data will be even larger.

### Fractional atomic coordinates and isotropic or equivalent isotropic displacement parameters (Å^2^)

**Table:**

	*x*	*y*	*z*	*U*_iso_*/*U*_eq_	Occ. (<1)
Cl1	0.73833 (6)	0.53784 (8)	0.11864 (4)	0.0566 (2)	
Cl2	0.73589 (8)	0.86623 (10)	0.29381 (4)	0.0697 (3)	
Cl3	0.75952 (8)	0.51754 (10)	0.41195 (6)	0.0931 (4)	
Cl4	0.75948 (8)	0.21696 (11)	0.22698 (4)	0.0719 (3)	
F1	0.5430 (3)	0.4344 (3)	0.16850 (18)	0.0600 (10)	0.50
F1A	0.5483 (5)	0.7210 (7)	0.3246 (2)	0.121 (2)	0.50
F2	0.9551 (3)	0.6350 (3)	0.3500 (2)	0.0771 (13)	0.50
F2A	0.9515 (4)	0.3907 (7)	0.2061 (2)	0.118 (2)	0.50
O1	0.91972 (15)	0.72821 (19)	0.18987 (9)	0.0473 (5)	
O2	0.57619 (15)	1.3317 (2)	−0.00869 (9)	0.0499 (5)	
O3	0.57623 (15)	0.3266 (2)	0.33521 (10)	0.0514 (5)	
O4	1.00724 (16)	−0.28448 (19)	0.48786 (9)	0.0499 (5)	
C1	0.6802 (2)	0.5932 (3)	0.17975 (13)	0.0418 (6)	
C2	0.5874 (2)	0.5310 (3)	0.20029 (16)	0.0554 (8)	
H2A	0.5557	0.4601	0.1804	0.066*	0.50
C3	0.5411 (3)	0.5714 (3)	0.24921 (17)	0.0611 (9)	
H3A	0.4776	0.5287	0.2634	0.073*	
C4	0.5875 (3)	0.6741 (4)	0.27727 (14)	0.0570 (9)	
H4A	0.5564	0.7018	0.3114	0.068*	0.50
C5	0.6787 (2)	0.7381 (3)	0.25700 (12)	0.0451 (7)	
C6	0.7264 (2)	0.6993 (2)	0.20724 (11)	0.0355 (5)	
C7	0.8245 (2)	0.7701 (2)	0.18319 (11)	0.0361 (6)	
C8	0.8000 (2)	0.8855 (2)	0.15059 (11)	0.0360 (5)	
H8A	0.8614	0.9377	0.1405	0.043*	
C9	0.6966 (2)	0.9225 (2)	0.13400 (11)	0.0357 (6)	
H9A	0.6362	0.8733	0.1475	0.043*	
C10	0.6665 (2)	1.0297 (2)	0.09763 (11)	0.0345 (5)	
C11	0.5534 (2)	1.0535 (3)	0.08407 (13)	0.0447 (7)	
H11A	0.4979	1.0002	0.0997	0.054*	
C12	0.5196 (2)	1.1523 (3)	0.04865 (13)	0.0470 (7)	
H12A	0.4420	1.1663	0.0400	0.056*	
C13	0.5993 (2)	1.2302 (3)	0.02593 (12)	0.0376 (6)	
C14	0.7130 (2)	1.2079 (3)	0.03816 (12)	0.0402 (6)	
H14A	0.7680	1.2607	0.0219	0.048*	
C15	0.7457 (2)	1.1101 (3)	0.07347 (12)	0.0386 (6)	
H15A	0.8235	1.0964	0.0818	0.046*	
C16	0.4637 (3)	1.3482 (3)	−0.02947 (16)	0.0602 (9)	
H16A	0.4596	1.4221	−0.0549	0.090*	
H16B	0.4148	1.3628	0.0022	0.090*	
H16C	0.4390	1.2712	−0.0500	0.090*	
C17	0.8124 (3)	0.4772 (3)	0.34698 (17)	0.0618 (9)	
C18	0.9025 (3)	0.5439 (3)	0.3250 (3)	0.0935 (18)	
H18A	0.9327	0.6150	0.3454	0.112*	0.50
C19	0.9488 (3)	0.5117 (5)	0.2757 (3)	0.104 (2)	
H19A	1.0113	0.5562	0.2616	0.125*	
C20	0.9013 (3)	0.4145 (4)	0.2488 (2)	0.0862 (15)	
H20A	0.9319	0.3914	0.2139	0.103*	0.50
C21	0.8131 (2)	0.3439 (3)	0.26580 (15)	0.0562 (9)	
C22	0.7669 (2)	0.3748 (3)	0.31718 (14)	0.0465 (7)	
C23	0.6745 (2)	0.2949 (3)	0.34163 (12)	0.0406 (6)	
C24	0.7103 (2)	0.1816 (3)	0.37319 (12)	0.0400 (6)	
H24A	0.6546	0.1310	0.3902	0.048*	
C25	0.8174 (2)	0.1453 (2)	0.37936 (11)	0.0360 (6)	
H25A	0.8709	0.1979	0.3617	0.043*	
C26	0.86199 (19)	0.0345 (2)	0.40993 (11)	0.0328 (5)	
C27	0.9691 (2)	−0.0094 (3)	0.39798 (11)	0.0367 (6)	
H27A	1.0117	0.0353	0.3713	0.044*	
C28	1.0141 (2)	−0.1161 (3)	0.42405 (12)	0.0418 (6)	
H28A	1.0864	−0.1458	0.4146	0.050*	
C29	0.9545 (2)	−0.1804 (2)	0.46389 (11)	0.0356 (5)	
C30	0.8491 (2)	−0.1371 (3)	0.47756 (12)	0.0404 (6)	
H30A	0.8083	−0.1801	0.5055	0.049*	
C31	0.8035 (2)	−0.0312 (3)	0.45043 (12)	0.0396 (6)	
H31A	0.7307	−0.0026	0.4596	0.048*	
C32	0.9572 (3)	−0.3425 (3)	0.53478 (13)	0.0508 (7)	
H32A	1.0056	−0.4118	0.5494	0.076*	
H32B	0.8835	−0.3773	0.5230	0.076*	
H32C	0.9479	−0.2783	0.5644	0.076*	

### Atomic displacement parameters (Å^2^)

**Table:**

	*U*^11^	*U*^22^	*U*^33^	*U*^12^	*U*^13^	*U*^23^
Cl1	0.0398 (4)	0.0599 (5)	0.0693 (5)	0.0048 (3)	−0.0089 (3)	−0.0187 (4)
Cl2	0.0707 (6)	0.0786 (6)	0.0601 (5)	−0.0014 (5)	0.0070 (4)	−0.0206 (5)
Cl3	0.0633 (6)	0.0740 (6)	0.1389 (10)	0.0131 (5)	−0.0371 (6)	−0.0454 (7)
Cl4	0.0612 (5)	0.0891 (7)	0.0663 (6)	0.0046 (5)	0.0130 (4)	0.0001 (5)
F1	0.0344 (17)	0.0391 (17)	0.106 (3)	−0.0165 (14)	−0.0007 (17)	−0.0054 (19)
F1A	0.111 (4)	0.154 (6)	0.102 (4)	0.008 (4)	0.048 (3)	0.035 (4)
F2	0.061 (2)	0.0393 (19)	0.129 (4)	−0.0120 (18)	−0.026 (2)	−0.012 (2)
F2A	0.077 (3)	0.191 (7)	0.086 (4)	0.000 (4)	0.016 (3)	0.056 (4)
O1	0.0286 (9)	0.0475 (11)	0.0655 (13)	0.0027 (8)	−0.0018 (8)	0.0070 (10)
O2	0.0358 (10)	0.0515 (12)	0.0622 (13)	0.0030 (9)	−0.0004 (9)	0.0201 (10)
O3	0.0288 (9)	0.0486 (11)	0.0764 (15)	0.0101 (9)	−0.0027 (9)	−0.0001 (11)
O4	0.0418 (10)	0.0481 (11)	0.0606 (13)	0.0136 (9)	0.0100 (9)	0.0170 (10)
C1	0.0287 (12)	0.0377 (14)	0.0585 (18)	0.0052 (11)	−0.0052 (11)	0.0057 (13)
C2	0.0306 (14)	0.0423 (16)	0.093 (3)	−0.0017 (12)	−0.0051 (15)	0.0131 (17)
C3	0.0391 (16)	0.056 (2)	0.089 (3)	0.0004 (15)	0.0112 (16)	0.0328 (19)
C4	0.0467 (17)	0.070 (2)	0.0554 (19)	0.0143 (16)	0.0148 (14)	0.0232 (17)
C5	0.0413 (15)	0.0473 (16)	0.0468 (16)	0.0065 (13)	−0.0002 (12)	0.0086 (13)
C6	0.0270 (12)	0.0346 (13)	0.0445 (14)	0.0044 (10)	−0.0036 (10)	0.0077 (11)
C7	0.0307 (12)	0.0364 (13)	0.0410 (14)	−0.0006 (10)	−0.0006 (10)	−0.0016 (11)
C8	0.0323 (12)	0.0354 (13)	0.0402 (14)	−0.0037 (10)	0.0011 (10)	0.0012 (11)
C9	0.0312 (12)	0.0336 (13)	0.0424 (14)	−0.0019 (10)	0.0034 (10)	0.0000 (11)
C10	0.0287 (11)	0.0339 (13)	0.0409 (14)	−0.0003 (10)	0.0013 (10)	0.0003 (11)
C11	0.0280 (12)	0.0464 (15)	0.0602 (18)	−0.0030 (11)	0.0065 (12)	0.0119 (14)
C12	0.0273 (12)	0.0516 (17)	0.0621 (19)	0.0025 (12)	0.0019 (12)	0.0139 (15)
C13	0.0318 (12)	0.0367 (13)	0.0442 (15)	0.0015 (11)	−0.0005 (10)	0.0028 (12)
C14	0.0308 (12)	0.0400 (14)	0.0499 (16)	−0.0041 (11)	0.0027 (11)	0.0063 (12)
C15	0.0274 (12)	0.0395 (14)	0.0486 (15)	−0.0014 (11)	−0.0029 (10)	0.0029 (12)
C16	0.0421 (16)	0.059 (2)	0.078 (2)	0.0068 (15)	−0.0109 (15)	0.0170 (18)
C17	0.0412 (16)	0.0400 (16)	0.102 (3)	0.0077 (13)	−0.0227 (17)	0.0070 (17)
C18	0.048 (2)	0.050 (2)	0.178 (5)	−0.0162 (18)	−0.052 (3)	0.047 (3)
C19	0.041 (2)	0.103 (4)	0.166 (5)	−0.017 (2)	−0.022 (3)	0.098 (4)
C20	0.0372 (18)	0.099 (3)	0.121 (4)	−0.001 (2)	−0.007 (2)	0.073 (3)
C21	0.0315 (14)	0.0593 (19)	0.078 (2)	0.0049 (13)	−0.0020 (14)	0.0289 (18)
C22	0.0287 (13)	0.0353 (14)	0.075 (2)	0.0052 (11)	−0.0088 (13)	0.0157 (14)
C23	0.0303 (13)	0.0363 (14)	0.0549 (17)	0.0047 (11)	−0.0011 (11)	−0.0043 (13)
C24	0.0309 (12)	0.0375 (13)	0.0518 (16)	0.0017 (11)	0.0052 (11)	0.0027 (12)
C25	0.0317 (12)	0.0324 (12)	0.0440 (15)	0.0010 (10)	0.0020 (10)	−0.0013 (11)
C26	0.0286 (11)	0.0312 (12)	0.0384 (14)	0.0008 (10)	−0.0013 (10)	−0.0047 (11)
C27	0.0290 (12)	0.0388 (13)	0.0425 (14)	0.0020 (10)	0.0054 (10)	0.0031 (12)
C28	0.0287 (12)	0.0472 (15)	0.0498 (16)	0.0081 (11)	0.0069 (11)	0.0037 (13)
C29	0.0326 (12)	0.0328 (12)	0.0412 (14)	0.0046 (10)	−0.0009 (10)	0.0014 (11)
C30	0.0361 (13)	0.0418 (14)	0.0439 (15)	0.0021 (12)	0.0094 (11)	0.0047 (12)
C31	0.0298 (12)	0.0402 (14)	0.0496 (16)	0.0057 (11)	0.0096 (11)	0.0015 (12)
C32	0.0507 (17)	0.0482 (17)	0.0536 (18)	0.0037 (14)	0.0040 (14)	0.0162 (14)

### Geometric parameters (Å, º)

**Table:**

Cl1—C1	1.732 (3)	C14—H14A	0.9500
Cl2—C5	1.724 (3)	C15—H15A	0.9500
Cl3—C17	1.741 (4)	C16—H16A	0.9800
Cl4—C21	1.723 (4)	C16—H16B	0.9800
O1—C7	1.220 (3)	C16—H16C	0.9800
O2—C13	1.363 (3)	C17—C22	1.381 (4)
O2—C16	1.419 (3)	C17—C18	1.398 (6)
O3—C23	1.219 (3)	C18—C19	1.354 (7)
O4—C29	1.369 (3)	C18—H18A	0.9500
O4—C32	1.420 (3)	C19—C20	1.315 (7)
C1—C2	1.387 (4)	C19—H19A	0.9500
C1—C6	1.388 (4)	C20—C21	1.359 (5)
C2—C3	1.372 (5)	C20—H20A	0.9500
C2—H2A	0.9500	C21—C22	1.396 (5)
C3—C4	1.368 (5)	C22—C23	1.516 (4)
C3—H3A	0.9500	C23—C24	1.456 (4)
C4—C5	1.378 (4)	C24—C25	1.333 (3)
C4—H4A	0.9500	C24—H24A	0.9500
C5—C6	1.391 (4)	C25—C26	1.455 (3)
C6—C7	1.514 (3)	C25—H25A	0.9500
C7—C8	1.456 (4)	C26—C31	1.391 (4)
C8—C9	1.335 (3)	C26—C27	1.395 (3)
C8—H8A	0.9500	C27—C28	1.373 (4)
C9—C10	1.450 (4)	C27—H27A	0.9500
C9—H9A	0.9500	C28—C29	1.379 (4)
C10—C11	1.393 (3)	C28—H28A	0.9500
C10—C15	1.403 (3)	C29—C30	1.384 (3)
C11—C12	1.381 (4)	C30—C31	1.381 (4)
C11—H11A	0.9500	C30—H30A	0.9500
C12—C13	1.376 (4)	C31—H31A	0.9500
C12—H12A	0.9500	C32—H32A	0.9800
C13—C14	1.393 (3)	C32—H32B	0.9800
C14—C15	1.368 (4)	C32—H32C	0.9800
			
C13—O2—C16	118.0 (2)	H16B—C16—H16C	109.5
C29—O4—C32	117.7 (2)	C22—C17—C18	119.0 (4)
C2—C1—C6	120.9 (3)	C22—C17—Cl3	119.5 (3)
C2—C1—Cl1	119.3 (2)	C18—C17—Cl3	121.4 (3)
C6—C1—Cl1	119.8 (2)	C19—C18—C17	122.9 (4)
C3—C2—C1	120.5 (3)	C19—C18—H18A	118.5
C3—C2—H2A	119.8	C17—C18—H18A	118.5
C1—C2—H2A	119.8	C20—C19—C18	115.5 (4)
C4—C3—C2	119.0 (3)	C20—C19—H19A	122.3
C4—C3—H3A	120.5	C18—C19—H19A	122.3
C2—C3—H3A	120.5	C19—C20—C21	126.7 (5)
C3—C4—C5	121.3 (3)	C19—C20—H20A	116.7
C3—C4—H4A	119.3	C21—C20—H20A	116.7
C5—C4—H4A	119.3	C20—C21—C22	118.0 (4)
C4—C5—C6	120.6 (3)	C20—C21—Cl4	122.2 (3)
C4—C5—Cl2	120.0 (3)	C22—C21—Cl4	119.7 (2)
C6—C5—Cl2	119.4 (2)	C17—C22—C21	117.9 (3)
C1—C6—C5	117.7 (2)	C17—C22—C23	120.4 (3)
C1—C6—C7	120.6 (2)	C21—C22—C23	121.6 (3)
C5—C6—C7	121.7 (2)	O3—C23—C24	123.1 (3)
O1—C7—C8	122.1 (2)	O3—C23—C22	120.6 (2)
O1—C7—C6	120.2 (2)	C24—C23—C22	116.3 (2)
C8—C7—C6	117.7 (2)	C25—C24—C23	123.2 (3)
C9—C8—C7	124.0 (2)	C25—C24—H24A	118.4
C9—C8—H8A	118.0	C23—C24—H24A	118.4
C7—C8—H8A	118.0	C24—C25—C26	127.5 (2)
C8—C9—C10	127.0 (2)	C24—C25—H25A	116.2
C8—C9—H9A	116.5	C26—C25—H25A	116.2
C10—C9—H9A	116.5	C31—C26—C27	117.7 (2)
C11—C10—C15	117.2 (2)	C31—C26—C25	123.7 (2)
C11—C10—C9	119.3 (2)	C27—C26—C25	118.6 (2)
C15—C10—C9	123.5 (2)	C28—C27—C26	121.2 (2)
C12—C11—C10	122.0 (2)	C28—C27—H27A	119.4
C12—C11—H11A	119.0	C26—C27—H27A	119.4
C10—C11—H11A	119.0	C27—C28—C29	120.1 (2)
C13—C12—C11	119.5 (2)	C27—C28—H28A	119.9
C13—C12—H12A	120.3	C29—C28—H28A	119.9
C11—C12—H12A	120.3	O4—C29—C28	115.6 (2)
O2—C13—C12	124.8 (2)	O4—C29—C30	124.6 (2)
O2—C13—C14	115.3 (2)	C28—C29—C30	119.9 (2)
C12—C13—C14	119.9 (2)	C31—C30—C29	119.7 (2)
C15—C14—C13	120.2 (2)	C31—C30—H30A	120.2
C15—C14—H14A	119.9	C29—C30—H30A	120.2
C13—C14—H14A	119.9	C30—C31—C26	121.3 (2)
C14—C15—C10	121.2 (2)	C30—C31—H31A	119.3
C14—C15—H15A	119.4	C26—C31—H31A	119.3
C10—C15—H15A	119.4	O4—C32—H32A	109.5
O2—C16—H16A	109.5	O4—C32—H32B	109.5
O2—C16—H16B	109.5	H32A—C32—H32B	109.5
H16A—C16—H16B	109.5	O4—C32—H32C	109.5
O2—C16—H16C	109.5	H32A—C32—H32C	109.5
H16A—C16—H16C	109.5	H32B—C32—H32C	109.5
			
C6—C1—C2—C3	−1.8 (4)	C22—C17—C18—C19	−0.9 (5)
Cl1—C1—C2—C3	178.8 (2)	Cl3—C17—C18—C19	177.1 (3)
C1—C2—C3—C4	0.2 (5)	C17—C18—C19—C20	1.7 (6)
C2—C3—C4—C5	0.8 (5)	C18—C19—C20—C21	−1.0 (6)
C3—C4—C5—C6	−0.3 (4)	C19—C20—C21—C22	−0.5 (5)
C3—C4—C5—Cl2	−179.4 (2)	C19—C20—C21—Cl4	−179.1 (3)
C2—C1—C6—C5	2.2 (4)	C18—C17—C22—C21	−0.7 (4)
Cl1—C1—C6—C5	−178.3 (2)	Cl3—C17—C22—C21	−178.7 (2)
C2—C1—C6—C7	−177.3 (2)	C18—C17—C22—C23	175.9 (3)
Cl1—C1—C6—C7	2.2 (3)	Cl3—C17—C22—C23	−2.2 (4)
C4—C5—C6—C1	−1.2 (4)	C20—C21—C22—C17	1.3 (4)
Cl2—C5—C6—C1	177.96 (19)	Cl4—C21—C22—C17	180.0 (2)
C4—C5—C6—C7	178.3 (3)	C20—C21—C22—C23	−175.1 (3)
Cl2—C5—C6—C7	−2.5 (3)	Cl4—C21—C22—C23	3.5 (4)
C1—C6—C7—O1	−79.7 (3)	C17—C22—C23—O3	86.1 (4)
C5—C6—C7—O1	100.9 (3)	C21—C22—C23—O3	−97.5 (3)
C1—C6—C7—C8	98.1 (3)	C17—C22—C23—C24	−93.0 (3)
C5—C6—C7—C8	−81.3 (3)	C21—C22—C23—C24	83.4 (3)
O1—C7—C8—C9	167.9 (3)	O3—C23—C24—C25	178.7 (3)
C6—C7—C8—C9	−9.8 (4)	C22—C23—C24—C25	−2.2 (4)
C7—C8—C9—C10	−174.4 (2)	C23—C24—C25—C26	179.9 (3)
C8—C9—C10—C11	179.2 (3)	C24—C25—C26—C31	−18.7 (4)
C8—C9—C10—C15	1.2 (4)	C24—C25—C26—C27	160.9 (3)
C15—C10—C11—C12	−0.4 (4)	C31—C26—C27—C28	1.8 (4)
C9—C10—C11—C12	−178.5 (3)	C25—C26—C27—C28	−177.7 (2)
C10—C11—C12—C13	−0.1 (5)	C26—C27—C28—C29	−1.6 (4)
C16—O2—C13—C12	−9.4 (4)	C32—O4—C29—C28	170.9 (3)
C16—O2—C13—C14	170.9 (3)	C32—O4—C29—C30	−8.3 (4)
C11—C12—C13—O2	−178.8 (3)	C27—C28—C29—O4	−179.2 (2)
C11—C12—C13—C14	0.9 (5)	C27—C28—C29—C30	0.0 (4)
O2—C13—C14—C15	178.6 (3)	O4—C29—C30—C31	−179.7 (3)
C12—C13—C14—C15	−1.1 (4)	C28—C29—C30—C31	1.2 (4)
C13—C14—C15—C10	0.6 (4)	C29—C30—C31—C26	−0.8 (4)
C11—C10—C15—C14	0.1 (4)	C27—C26—C31—C30	−0.6 (4)
C9—C10—C15—C14	178.2 (3)	C25—C26—C31—C30	178.9 (3)

### Hydrogen-bond geometry (Å, º)

**Table:**

*D*—H···*A*	*D*—H	H···*A*	*D*···*A*	*D*—H···*A*
C2—H2*A*···F1*A*^i^	0.95	2.79	3.657 (7)	153
C4—H4*A*···F1^ii^	0.95	2.75	3.410 (5)	127
C11—H11*A*···O3^ii^	0.95	2.56	3.451 (3)	156

Symmetry codes: (i) −*x*+1, *y*−1/2, −*z*+1/2; (ii) −*x*+1, *y*+1/2, −*z*+1/2.

### Selected geometric parmeters (Å): Cg···Cg π stacking interactions, Cg1, Cg3 are the centroids of rings C1—C6 and C17—C22 [Symmetry codes: (i) x, y, z]

**Table:**

*Cg*I···*Cg*J	Cg···Cg (Å)	CgI Perp (Å)	Cgj Perp (Å)
Cg1···Cg3^i^	3.650 (2)	3.620	3.604
